# Supplementary material for: Characterization of the Astrocyte Calcium Response to Norepinephrine in the Ventral Tegmental Area
Source: Cells. 2024 Dec 30;14(1):24. doi: 10.3390/cells14010024 (PMC11720743; doi:10.3390/cells14010024)
Supplement: Supplementary file 1 [file cells-14-00024-s001.zip › cells-3306004-supplementary.pdf]

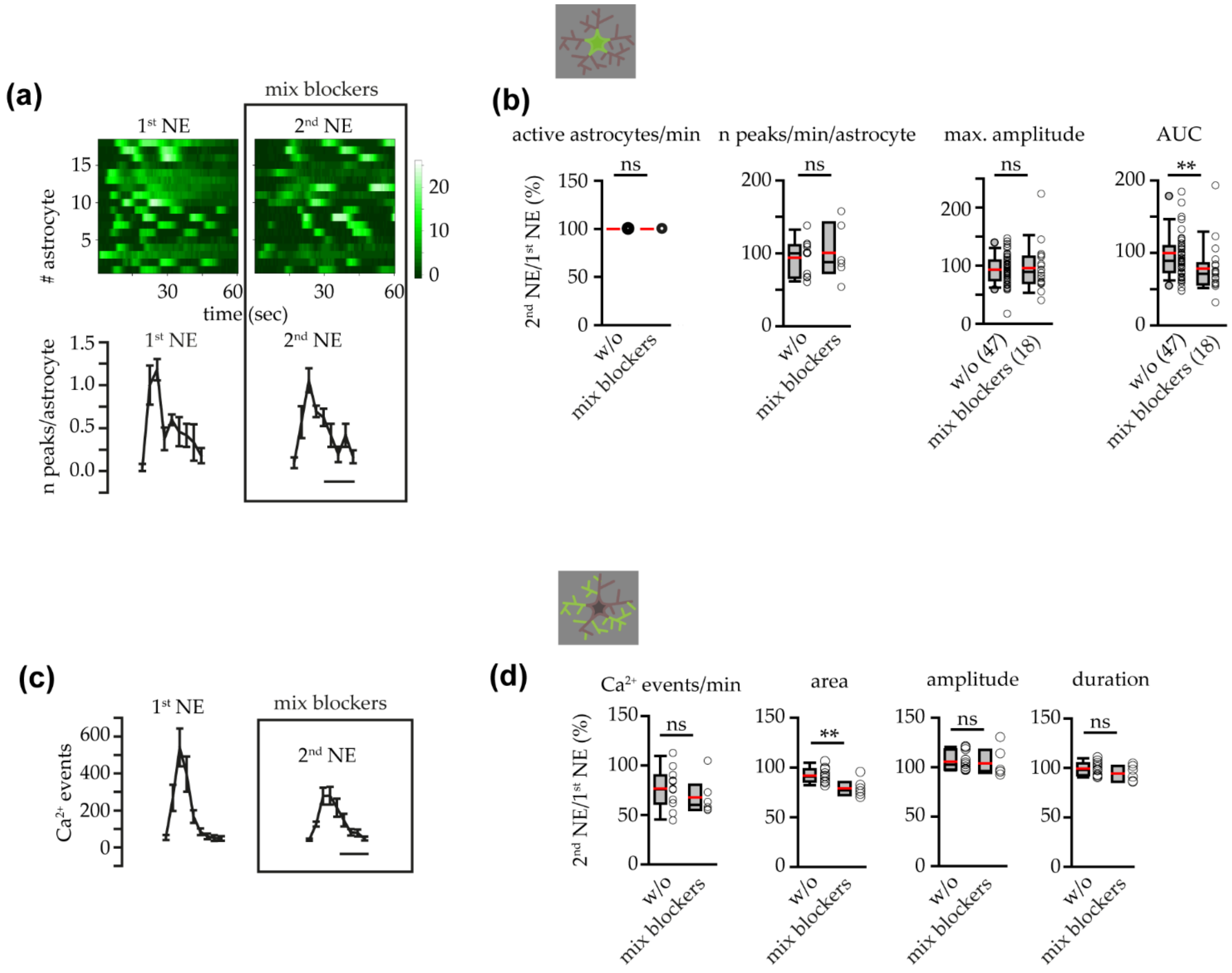

Supplemental Figure S1. VTA astrocyte Ca^{2+} responses to NE in the presence of a mix of blockers. (**a**) Upper panel, heatmaps showing the $\Delta F/F_0$ in the soma of all astrocytes challenged with NE in the absence and in the presence of a mix of blockers. Lower panel, time course of the mean number of somatic Ca^{2+} peaks per astrocyte under different conditions (n = 6 slices with 18 astrocytes from 4 mice). Scale bar, 1 min. (**b**) Box and whisker plots reporting the somatic NE response of VTA astrocytes in the absence and in the presence of a mix of blockers. (slices: w/o antagonist, n = 11; mix blockers, n = 6; astrocytes: w/o antagonist, n = 47; mix blockers, n = 18; frequency of somatic peaks per astrocyte, two-tailed t test ; Mann-Whitney rank sum for the other analyzed parameters). (**c**) Time course of the mean number of astropile Ca^{2+} events during NE application in the absence or presence of a mix of blockers (n = 6 slices). Scale bar, 1 min. (**d**) Box and whisker plots showing the percentages of the frequency, mean area, mean amplitude and mean duration of the astropile Ca^{2+} events observed with NE, in different conditions (w/o antagonist, n = 11; mix of blockers, n = 6; mean amplitude, Mann-Whitney rank sum test; two-tailed t test for comparisons of the other parameters).

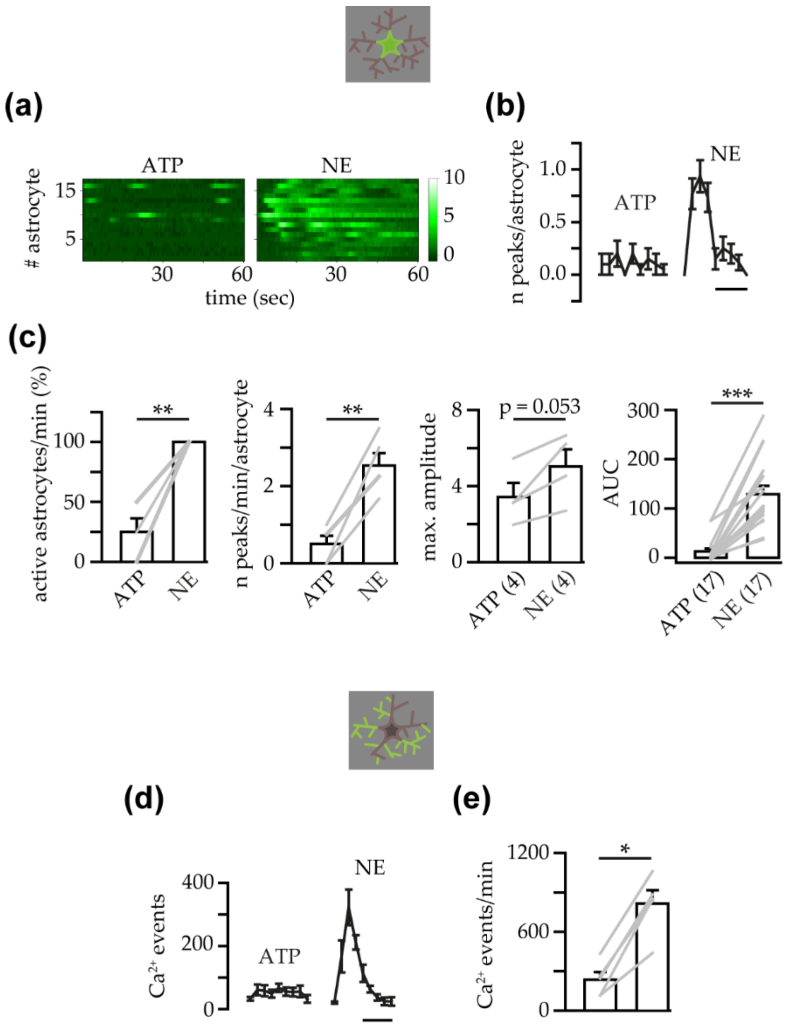

Supplemental Figure S2. VTA astrocyte Ca^{2+} activity in response to ATP and NE. Mice used in these experiments were injected with 0.6 µl of the AAV to express GCaMP6f in astrocytes. (**a**) Heatmaps of the $\Delta F/F_0$ in the soma of all astrocytes analyzed during the challenging with ATP (100 µM) or NE (10 µM). (**b**) Time course of the mean number of somatic Ca^{2+} peaks per astrocyte in the different experimental conditions (n = 5 slices with 17 astrocytes from 3 mice). Scale bar, 1 min. (**c**) Bar charts showing the activity of VTA astrocyte somata in the presence of ATP or NE (two-tailed paired t test). (**d**) Time course of the mean number of astropile Ca^{2+} events in the different experimental conditions. Scale bar, 1 min. (**e**) Bar charts showing the activity of VTA astrocytes at processes in the presence of ATP or NE (two-tailed paired t test).
